# Enhanced visual perception with occipital transcranial magnetic stimulation

**DOI:** 10.1111/j.1460-9568.2011.07814.x

**Published:** 2011-10

**Authors:** Manon Mulckhuyse, Todd A Kelley, Jan Theeuwes, Vincent Walsh, Nilli Lavie

**Affiliations:** 1 Department of Cognitive Psychology, Vrije UniversiteitAmsterdam, The Netherlands; 2 Department of Experimental Clinical and Health Psychology, Ghent UniversityHenri Dunantlaan 2, B-9000 Ghent, Belgium; 3 Department of Psychology, University College of LondonLondon, UK; 4 Institute of Cognitive Neuroscience, University College of LondonLondon, UK; 5 Center for Mind and Brain, University of CaliforniaDavis, CA, USA

**Keywords:** alpha-band, cortical excitability, human, occipital transcranial magnetic stimulation, spatial cueing, visual perception

## Abstract

Transcranial magnetic stimulation (TMS) over the occipital pole can produce an illusory percept of a light flash (or ‘phosphene’), suggesting an excitatory effect. Whereas previous reported effects produced by single-pulse occipital pole TMS are typically disruptive, here we report the first demonstration of a location-specific facilitatory effect on visual perception in humans. Observers performed a spatial cueing orientation discrimination task. An orientation target was presented in one of two peripheral placeholders. A single pulse below the phosphene threshold applied to the occipital pole 150 or 200 ms before stimulus onset was found to facilitate target discrimination in the contralateral compared with the ipsilateral visual field. At the 150-ms time window contralateral TMS also amplified cueing effects, increasing both facilitation effects for valid cues and interference effects for invalid cues. These results are the first to show location-specific enhanced visual perception with single-pulse occipital pole stimulation prior to stimulus presentation, suggesting that occipital stimulation can enhance the excitability of visual cortex to subsequent perception.

## Introduction

Research concerning the underlying neural mechanisms of visual perception has often used transcranial magnetic stimulation (TMS) as a method to disrupt perception (e.g. Amassian *et al.*, 1989, 1998; Beckers & Homberg, 1991; Kammer *et al.*, 2005b; Pascual-Leone & Walsh, 2001; Ro *et al.*, 2003). A classic example of TMS-induced interference with visual perception is the seminal study by Amassian *et al.* (1989). This study investigated the time window in which early visual brain areas are essential for vision by applying single-pulse TMS to occipital cortex while observers identified briefly presented letters. The results showed that single-pulse TMS disrupts visual processing between 80 and 100 ms after stimulus onset.

In contrast to the disruptive effects on visual perception, single-pulse TMS over occipital cortex can also induce the percept of a light flash, i.e. phosphene (Cowey & Walsh, 2000; Kammer, 1999; Meyer *et al.*, 1991). TMS induction of phosphenes is typically considered to be excitatory (e.g. Aurora & Welch, 1998; Battelli *et al.*, 2002; Boroojerdi *et al.*, 2000; Muggleton *et al.*, 2008). Indeed, studies combining TMS and electroencephalography demonstrated a reduced alpha-band activity in posterior sites contralateral to the occipital TMS side, suggesting location-specific enhanced visual cortex excitability (Romei *et al.*, 2008a). These findings suggest that under some circumstances occipital stimulation can sensitize the visual cortex and may thus facilitate visual perception, but this has not previously been demonstrated.

We sought to address this in the present study. We used a spatial cueing paradigm using luminance cues in an orientation discrimination task and applied sub-threshold single-pulse TMS over the occipital pole targeting primary visual cortex (V1). The use of spatial cueing made it possible to also examine whether occipital pole stimulation could compete with the cueing effects of a visual stimulus (the luminance cue). A single pulse of TMS administered at sub-threshold intensity (below phosphenes threshold) was applied over the occipital pole at −950, −350, −200 and −150 ms relative to target onset (these corresponded to −750, −150, 0 and +50 ms relative to cue onset, respectively).

If occipital TMS can enhance visual perception, then we should find facilitation effects for targets presented in the visual field contralateral to the stimulated hemisphere (compared with ipsilateral targets). In addition, we should find enhanced cueing effects for the luminance cues presented contralateral to the stimulated hemisphere and reduced cueing effects for cues presented ipsilateral to the stimulated hemisphere. Note that these effects can be clearly distinguished from any general non-TMS-specific spatial cueing effects that may be due to the noise of the coil cueing attention to the coil side. Any such effects would be observed on stimuli presented ipsilateral of the stimulated TMS side, whereas effects due to the stimulation itself are expected to affect stimuli contralateral of the stimulated TMS side.

## Materials and methods

### Participants

Eight paid participants (five female, aged between 21 and 38 years) performed the experiment. All participants had previous experience participating in TMS experiments and gave informed consent before participation. Seven participants were naive to the objective of the study, and the other participant was M.M. The study was approved by the UCL ethics committee.

### Apparatus

E-Prime software (Psychology Software Tools) was used for stimulus presentation, data recording and to control the TMS timing. Displays were presented on a monitor with a resolution of 1024 × 768 pixels and a 60-Hz refresh rate. The distance between monitor and chin rest was 50 cm. Single-pulse TMS was delivered using a 70-mm figure-of-eight coil with a Magstim Rapid stimulator (Magstim Company). The experiment was conducted in a dimly lit room.

### TMS procedure

The experiment consisted of two sessions. In one session (long trials), TMS was applied at 950 or 350 ms before target onset (750 or 150 ms before cue onset) and in the other session (short trials) TMS was applied at 200 or 150 ms before target onset (0 or 50 ms after cue onset). The sessions were counterbalanced across subjects. Before a session started phosphenes were localized for each participant. Participants wore swimming caps in order to mark the stimulated site on the head. Localization was conducted by starting 2 cm dorsal and 0.5 cm to the left from the inion. Every participant started with single pulses of 75% of maximum output of the stimulator. The coil handle pointed horizontally to the right for all participants. If no phosphene was observed, the coil was moved slightly around the starting point. Once participants reported a lateralized phosphene, they were asked to fixate a dimly lit fixation point in the centre of the monitor and to point the curser in the middle of the phosphene. By clicking in the middle of the phosphene, the *x* and *y* coordinates relative to the fixation point were recorded. The TMS site was marked with a sticker on the head of the participant.

Six participants reported seeing phosphenes at 75% of maximum output and two participants at 85%. Furthermore, in two of the eight participants no phosphenes were elicited with the left hemisphere stimulation, and therefore for these the right hemisphere was stimulated (which resulted in phosphenes in the left visual field). All reported phosphenes were elicited in the lower visual field contralateral to the stimulated hemisphere. During the task, TMS was delivered between 60 and 57% of stimulator output to produce sub-threshold intensity. At these outputs, none of the participants reported seeing phosphenes.

### Stimuli and experimental procedure

All stimuli were presented on a grey background (4.2 cd/m^2^). Participants initiated a trial by pressing the space bar. After pressing the space bar, a black fixation cross and two placeholders, one to the left and one to the right of fixation, were presented. The distance between fixation and placeholder was dependent on the location of the reported phosphene. The previously recorded *x* and *y* coordinates were used to align the visual stimulus position, i.e. one of the placeholders, with the TMS-induced phosphene position. The other placeholder was moved to the opposite symmetrical location. The mean horizontal distance of the centre of the placeholders and fixation was 7.4° and mean vertical distance between fixation and the centre of the placeholders was 2.3°.

The placeholders consisted of two concentric open squares, an inner one (dark grey, 0.14 cd/m^2^) and an outer one (light grey, 5.4 cd/m^2^) of 2.3° on each side. In order to cue the participants, the outer square was illuminated (46 cd/m^2^) for one refresh rate (17 ms). Two hundred milliseconds after cue onset, the target was presented for 80 ms. The target consisted of a small green (8.99 cd/m^2^) horizontal or vertical line segment of 0.6° that was presented within either the left or the right placeholder. Participants were asked to keep their eyes fixated at the fixation point and press as fast as they could, with their right hand on the numeric keyboard, a ‘1’ when the line segment was vertical or a ‘2’ when the line segment was horizontal.

**Fig. 1:**
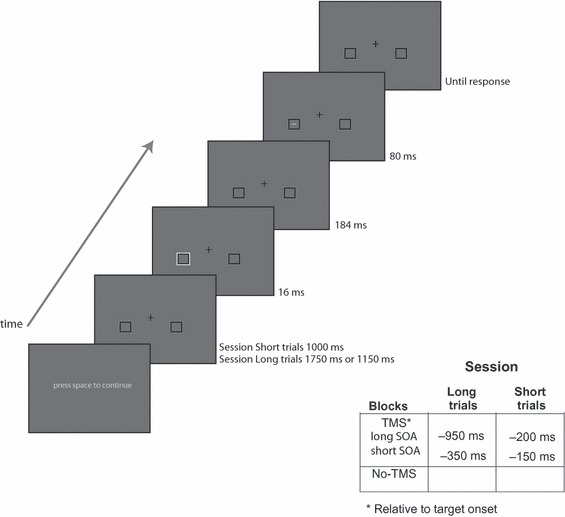
An example trial sequence showing a valid cue. Placeholders were presented for 1000 ms when TMS was applied 200 or 150 ms (i.e. 0 or 50 ms after cue onset) before target onset. When TMS was applied 950 or 350 ms before target onset (i.e. 750 or 150 ms before cue onset), the placeholders were presented for 1750 or 1150 ms, respectively. The cue consisted of the brightening of one of the placeholders for one refresh rate. Two hundred milliseconds after cue onset, the target, a small horizontal or vertical line, was presented for 80 ms. Participants responded to the line orientation of the target.

Each session consisted of 15 blocks. Each block consisted of 32 trials in which the cue was either valid (in 25% of trials the cue was presented at the same location as the target), invalid (in 25% of trials the cue was presented at the opposite location to the target), neutral (in 25% of trials the cue was presented at both locations simultaneously) or absent (in 25% of trials no cue was presented). The target could appear with equal probability on the left (50%) or the right (50%) of fixation and was either vertical (50%) or horizontal (50%). These conditions were randomly presented during a block. In three blocks, no TMS was delivered. These blocks were interleaved between every four TMS blocks. Half of the participants started with a no TMS block and half of the participants ended with a no TMS block. In the long trials session, TMS was delivered either 950 or 350 ms before target onset (i.e. 750 or 150 ms before cue onset) and in the short trials session, TMS was delivered at 200 or 150 ms before target onset (i.e. 0 or 50 after cue onset). These TMS timings were randomly applied in a session. Figure 1 presents the sequence of a trial.

## Results

Error rates were low (< 3%) and did not significantly vary between the experimental conditions. The analyses were based on the reaction time (RT) for the correct responses. RTs above or below 2.5 standard deviations of the mean (< 3%) were considered outliers and eliminated from any analyses.

### TMS effects on target processing

**Fig. 2:**
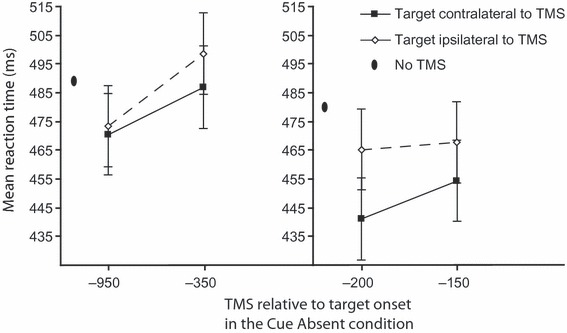
Mean reaction times in the cue-absent condition as a function of TMS timing relative to target onset for the long trials session (left panel) and the short trials session (right panel). Solid lines depict the mean reaction time when the target was presented in the visual field contralateral to TMS and dotted lines depict the mean reaction time when the target was presented in the visual field ipsilateral to TMS. Error bars represent normalized standard errors (Loftus & Masson, 1994). For each session, the mean reaction time in the no TMS condition is depicted as an oval.

To examine the effect of TMS on the target stimulus processing we conducted repeated-measures anova of the RTs in the cue-absent conditions with session (long trials, short trials), timing [long stimulus onset asynchrony (SOA), short SOA] and TMS side (contralateral to target, ipsilateral to target) as factors. The results are shown in Fig. 2. This anova revealed main effects of session (*F*_1,7_ = 7.27, *P* < 0.05), timing (*F*_1,7_ = 9.88, *P* < 0.05) and TMS side (*F*_1,7_ = 5.59, *P* = 0.05) and a three-way interaction between session, timing and TMS side (*F*_1,7_ = 7.26, *P* < 0.05). This interaction reflected differing effects of TMS and timing in the different sessions. In the long trials session, there was only a main effect of timing, RT was shorter in the 950-ms than in the 350-ms SOA (*F*_1,7_ = 18.71, *P <* 0.01) but there was no effect of TMS side (*F* < 1) and no interaction (*P* > 0.1) The effect of timing most likely reflects a general ‘warning effect’ as it did not interact with TMS side and warning effects typically benefit from a longer period between the warning signal and the target stimulus (e.g. Los and Van den Heuvel, 2001).

In the short trials session, there was a main effect of TMS side, and RT was shorter to targets contralateral to the TMS side compared with ipsilateral targets (*F*_1,7_ = 10.97, *P <* 0.05). This effect did not interact with timing (*P* > 0.19), nor was there a main effect of timing (*P* > 0.29). These results indicate that sub-threshold TMS enhances visual processing in the contralateral visual field when applied to occipital cortex 200 or 150 ms before stimulus onset.

A further anova with factors of TMS blocks (first third, last third), timing (long SOA, short SOA) and TMS side (contralateral to target, ipsilateral to target) revealed no interactions with blocks (all *F* < 1). Thus, the TMS effects we report were found across the duration of the experiment.

The effect of TMS on target processing was further compared with the no TMS conditions in anovas with the factor of TMS (contralateral to target, ipsilateral to target, no TMS) in each of the timings separately (because timing was not a factor in the no TMS condition). These revealed no effect of TMS when TMS was applied 950 ms (*P* = 0.1) or 350 ms (*P* = 0.2) before target onset. When TMS was applied 200 and 150 ms before target onset, there was an effect of TMS (*F*_2,14_ = 11.64, *P <* 0.01; *F*_2,14_ = 14.69, *P <* 0.01, respectively), reflecting shorter reaction times in the TMS conditions relative to the no TMS condition. Further *F* contrasts confirmed a significant facilitation for the contralateral TMS compared with no TMS at both time windows (both *P* < 0.01) and a small trend of marginal significance (*P* = 0.05) for facilitation with ipsilateral TMS compared with no TMS in the 150-ms time window. There was no effect of ipsilateral TMS in the 200-ms time window (*P* = 0.1).

### TMS effects on cue processing

**Fig. 3:**
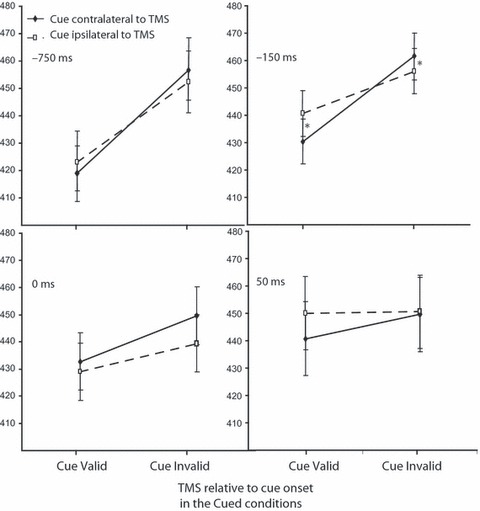
Mean reaction times for each different TMS timing relative to cue onset as a function of cue validity. Filled diamonds with solid lines depict mean reaction time when the cue was presented in the visual field contralateral to TMS, and open squares with dashed lines when the cue was presented in the visual field ipsilateral to TMS. Error bars represent normalized standard errors (Loftus & Masson, 1994). The asterisks at −150 ms before cue onset indicate significant differences between contralateral and ipsilateral TMS relative to cue presentation for the valid and invalid condition.

To examine the effect of TMS side and timing of the TMS pulse on cue processing we conducted repeated-measures anova of the RTs in the cue conditions with session (long trials, short trials), timing (long SOA, short SOA), TMS side (contralateral to cue, ipsilateral to cue) and cue-validity (valid, invalid) as factors.* The results are shown in Figure 3. This anova revealed a main effect of cue validity and timing: RT was shorter in the valid compared with the invalid cue conditions, (*F*_1,7_ = 61.4, *P* < 0.01) and in the long compared with the short SOA conditions (*F*_1,7_ = 7.46, *P* < 0.05). There were no effects for session (*F* < 1) or TMS side (*F* < 1) and no four-way or three-way interactions (all *P* > 0.28). There were also no two-way interactions (*P* > 0.19 for all) except for an interaction between session and cue validity (*F*_1,7_ = 41.09, *P* < 0.01), indicating that cue validity had a larger effect in the long trials session than in the short trials session.

The larger cueing effect in the long session appears to be due to an enhancement of the cueing effect in the TMS conditions (*M* = 457 ms in the invalid cue conditions and *M* = 428 ms in the valid cue conditions) compared with the no TMS conditions (*M* = 438 ms for the invalid cue condition and *M* = 428 ms for the valid cue condition). By contrast, in the short session, cueing effects were not enhanced due to TMS (*M* = 457 ms in the invalid cue conditions and *M* = 428 ms in the valid cue conditions) compared with the no TMS conditions (*M* =460 ms for the invalid cue condition and *M* = 430 for the valid cue condition). This pattern was further clarified in the following anovas with the factors of cue conditions (valid, invalid) and TMS (contralateral to cue, ipsilateral to cue, no TMS) in each of the TMS timings.†

### 750 ms before cue onset

There was a main effect of cue validity (*F*_1,7_ = 30.35, *P <* 0.01), indicating slower RTs in the invalid compared with the valid conditions. TMS condition had no main effect (*F* < 1) and there was no interaction (*F*_2,14_ = 3.09, *P =* 0.08).

### 150 ms before cue onset

There was a main effect of TMS condition (*F*_2,14_ = 11.59, *P <* 0.01). RTs were slower in both of the TMS conditions compared with the no TMS condition (both *P* < 0.05). There was also a main effect of cue validity (*F*_1,7_ = 23.6, *P <* 0.01), indicating slower responses in the invalid compared with the valid conditions and an interaction between TMS condition and cue validity (*F*_2,14_ = 3.8, *P <* 0.05). The interaction reflected that whereas in the invalid cue conditions contralateral TMS slowed responses (compared with both the ipsilateral condition (*t*_7_ = 1.92, *P* < 0.05, one-tailed), and the no TMS condition (*t*_7_ = 4.92, *P* < 0.01); in the valid cue condition contralateral TMS facilitated RT compared with the ipsilateral condition (*t*_7_ = 1.76, *P* = 0.06, one-tailed) but not with the no TMS condition (*t* < 1).

A further anova with factors of TMS blocks (first third, last third), cue validity (valid, invalid) and TMS (contralateral to cue, ipsilateral to cue) revealed no interaction with blocks (all *F* < 1). Thus this effect was found across the duration of the experiment.

### 0 ms at and 50 ms after cue onset

There were only main effects of cue validity (*P* < 0.01 for both), indicating slower responses in the invalid compared with the valid conditions. There were no effects for TMS condition or interactions (all *P* > 0.16).

## Discussion

The present results demonstrate that single-pulse TMS administered at a sub-threshold intensity to the occipital pole contralateral to the stimulus can facilitate the visual processing both for orientation targets (at the time windows of 200 and 150 ms prior to the stimulus onset) and for luminance cues at the time window of 150 ms prior to the stimulus onset. These results demonstrate a location-specific facilitation of visual perception with single-pulse occipital pole stimulation prior to the stimulus presentation.

These effects concur with previous demonstrations of the excitatory effects TMS has on visual cortex activity (Aurora & Welch, 1998; Battelli *et al.*, 2002; Boroojerdi *et al.*, 2000) and complement more recent findings (Romei *et al.*, 2008a) that a single-pulse TMS over the occipital pole involves a decrease in alpha-band activity in the contralateral field. Reduced alpha activity in posterior occipital cortex is highly correlated with improved perception (e.g. Romei *et al.*, 2008b; for a review see Thut & Miniussi, 2009). Indeed, the extent to which the TMS pulse produced a phosphene sensation was dependent on the alpha-band level of activity (Romei *et al.*, 2008a).

The contrast between the present findings of enhanced visual perception and the large number of previous reports on the disruptive effects of single-pulse TMS on vision (e.g. Amassian *et al.*, 1998; Harris *et al.*, 2008; Romei *et al.*, 2007; Thut *et al.*, 2003) may be attributed to a different timing or different stimulation intensity. For example, previous studies specifically investigated the time window in which occipital TMS disrupts visual perception after stimulus onset, whereas in the current TMS study we investigated the time window in which occipital TMS affects visual perception before stimulus onset. Furthermore, previous studies used far higher stimulation intensities (typically over 10–20% above phosphene threshold, sometimes as high as 90 or 100% of stimulator machine output (e.g. Amassian *et al.*, 1998) compared with the sub-threshold intensities used here as well as in previous TMS demonstrations of a facilitation effect measured with the threshold for phosphene induction (e.g. Ramos-Estebanez *et al.*, 2007; Silvanto *et al.*, 2005). Studies examining the underlying mechanisms involved in visual suppression by TMS showed that intensities are higher for suppression compared with phosphene perception (Kammer *et al.*, 2005b; Kastner *et al.*, 1998) and, additionally, higher TMS intensities increase the threshold for orientation discrimination (Kammer *et al.*, 2005a).

In line with the state dependency effects of TMS (e.g. Silvanto *et al.*, 2008b; Silvanto & Pascual-Leone, 2008), sub-threshold intensities may be more effective in sensitizing visual cortex to further retinal input. In addition it is important to bear in mind that the present findings were obtained within an attentional cueing paradigm. For instance, although the facilitation effects established for the target processing were found on those trials in which a cue was absent, these cue-absent trials were intermixed in random with cue-present trials. Thus it remains possible that the effect of facilitation in visual processing we established depends on the particular imbalance in states of neural activity produced by cueing. Unlike previous state dependency studies (Silvanto *et al.*, 2007,2008a) the effects found here do not suggest a particular state of activity is necessary for TMS facilitation. For instance, in a previous study (Cattaneo *et al.*, 2008) using the priming paradigm, an occipital single TMS pulse had only facilitated letter (consonant vs. vowel) discrimination for unprimed letters but TMS had no effect on primed letters. In contrast, our findings showed enhanced processing for both invalid cue condition (in which the processing state is arguably more similar to an unprimed condition) and valid cue condition (in which the processing state is arguably more similar to a primed condition). One potentially important difference between the present and previous findings is that our facilitory effects were location specific (found for contralateral vs. ipsilateral stimulation) and thus clearly indicative of a change in stimulus processing that only affected the stimulated occipital site. In contrast, the effects of TMS on priming were found in comparison of left parietal TMS with a no TMS condition.

Nevertheless the overall imbalance in activation induced by cueing may be necessary for the location-specific facilitation effect we report. Indeed, recent work on state dependency effects has established that subthreshold occipital TMS can enhance target processing (e.g. spatial position discrimination) only if the perceptual judgement was preceded with visual short-term memory (VSTM) maintenance of stimuli related to the target (a clock face, Cattaneo *et al.*, 2009). In the absence of the VSTM task, target detection was not facilitated with TMS.

In addition, it is worth considering the nature of the perceptual task and visual stimuli we have used. It is plausible that the processing involved in the perception of luminance cues and in the simple vertical vs. horizontal orientation discrimination task could benefit from the visual cortex excitation with occipital pole TMS. More subtle perceptual discriminations may instead be disrupted. In addition, stimulation of other parts of visual cortex (over MT/V5) may prove only to facilitate retinal inputs for which that part of visual cortex is selective for (e.g. motion input in the latter example). These are promising avenues for future research.

## Footnotes

* The neutral cue conditions (where a cue was presented on both sides) were not included as a factor in these anovas as TMS side cannot be assigned for these conditions. The mean (*M*) neutral cue RTs (*M* = 427 ms in the 750-ms SOA, *M* = 444 ms in the 150-ms SOA; *M* = 443 ms in the 0-ms SOA and *M* = 439 ms in the 50-ms SOA) were shorter than the invalid cue RT in all conditions, indicating a clear cost with the invalid cue. In the −150 ms time window with the contralateral stimulation the neutral cue RT was significantly shorter than the invalid cue RT (*t*_7_ = 4.12, *P* < 0.01), and longer than the valid cue RT (*t*_7_ = 2.43, *P* < 0.05) thus confirming that the contralateral TMS enhanced both cueing effects of cost and the benefit.

† Although the three-way interaction between timing, cueing and TMS was not significant, the finding that TMS facilitates target stimulus processing at the 150- to 200-ms time windows provides *a prior* hypothesis concerning the effects of TMS in the corresponding TMS to cue onset (150 ms) time window. This hypothesis justifies the conduct of planned comparisons for the effect of TMS on cueing in each of the time windows, even in the absence of a significant three-way interaction in the omnibus anova. We note also that a three-way interaction has an inherently weak power to reveal the particular pattern predicted on the basis of the target processing data, namely that an effect of TMS on cueing will be found in just one of the four levels of the timing factor (i.e. just in the 150-ms time window as predicted).

## Abbreviations

RT: reaction time
TMS: transcranial magnetic stimulation
VSTM: visual short-term memory
